# Sparsity-Based Recovery of Three-Dimensional Photoacoustic Images from Compressed Single-Shot Optical Detection

**DOI:** 10.3390/jimaging7100201

**Published:** 2021-10-02

**Authors:** Dylan Green, Anne Gelb, Geoffrey P. Luke

**Affiliations:** 1 Department of Mathematics, Dartmouth College, Hanover, NH 03755, USA; Dylan.P.Green.GR@dartmouth.edu (D.G.); AnneGelb@math.dartmouth.edu (A.G.); 2 Thayer School of Engineering, Dartmouth College, Hanover, NH 03755, USA

**Keywords:** photoacoustic imaging, compressed sensing, inverse problems, compressed ultrafast photography

## Abstract

Photoacoustic (PA) imaging combines optical excitation with ultrasonic detection to achieve high-resolution imaging of biological samples. A high-energy pulsed laser is often used for imaging at multi-centimeter depths in tissue. These lasers typically have a low pulse repetition rate, so to acquire images in real-time, only one pulse of the laser can be used per image. This single pulse necessitates the use of many individual detectors and receive electronics to adequately record the resulting acoustic waves and form an image. Such requirements make many PA imaging systems both costly and complex. This investigation proposes and models a method of volumetric PA imaging using a state-of-the-art compressed sensing approach to achieve real-time acquisition of the initial pressure distribution (IPD) at a reduced level of cost and complexity. In particular, a single exposure of an optical image sensor is used to capture an entire Fabry–Pérot interferometric acoustic sensor. Time resolved encoding as achieved through spatial sweeping with a galvanometer. This optical system further makes use of a random binary mask to set a predetermined subset of pixels to zero, thus enabling recovery of the time-resolved signals. The Two-Step Iterative Shrinking and Thresholding algorithm is used to reconstruct the IPD, harnessing the sparsity naturally occurring in the IPD as well as the additional structure provided by the binary mask. We conduct experiments on simulated data and analyze the performance of our new approach.

## 1. Introduction

Photoacoustic (PA) imaging provides a method of in vivo non-invasive and high-resolution molecular imaging at centimeter depth scales [1,2,3,4]. In PA imaging, biological tissue is irradiated by a pulsed laser. The absorption of the laser by endogenous or exogenous chromophores induces a local increase in temperature, which in turn causes a pressure rise through thermoelastic expansion of the tissue. Ultrasound receivers placed at the surface of the tissue detect the resulting acoustic waves. Images of the optical absorption can be reconstructed by solving acoustic and optical inverse problems [5]. While high-speed data acquisition is possible with PA imaging, the multichannel data acquisition systems that are available to record this data are expensive [6]. The detection of PA signals is most commonly accomplished using digital-to-analog converters recording the voltage connected to piezoelectric transducers, which drastically increase the complexity of the imaging system [7]. A well-established alternative to this is a method for optical interferometric detection which utilizes a Fabry–Pérot etalon (FPE) [8,9,10]. The FPE exploits the resonant interference of an interrogating continuous wave laser between two reflecting surfaces to provide both sensitive detection and large acoustic bandwidth.

A major limitation to using a FPE for PA signal detection, however, is that detection is performed only at a single point on the etalon. In practice, the detection laser must be raster scanned along the surface of the etalon to acquire a volumetric image of the tissue. Thus, the PA signal generating laser must be shot for each position in the raster scan to produce the photoacoustic signal, making the temporal resolution highly dependent on the pulse repetition rate of the laser. This dependence makes the imaging system prone to motion artifacts and unable to capture fast dynamic processes.

In order to improve the temporal resolution of the imaging system, recent work has been done in which a FPE was imaged onto a scrambled Hadamard pattern over multiple sequential measurements [11,12]. Though the main features of imaging phantoms used were successfully recovered at compression rates as low as 10%, multiple acquisitions of data were necessary, and thus the total data acquisition time was still much longer than pulse repetition rate of the laser.

Here, FPE-based PA image detection is combined with compressed ultrafast photography to further improve the temporal resolution. Compressed ultrafast photography applies core compressed sensing principles to acquire a sequence of images at a high rate using a single exposure of a camera [13]. In the original work, a random binary mask is applied to the image, and a streak camera is then used to scan the image quickly across a sensor [14]. The mask in conjunction with the streak camera provide enough structure such that the original time-resolved image sequence can be reconstructed. This has enabled reconstruction of 980-frame videos with framerates achieving $7\times 10^{13}$ frames per second [15]. In applications that do not require such extreme framerates, the streak camera can be replaced with a low-cost galvanometer to achieve the spatial shifting of the image [16].

In this work, we design and simulate an optical system that is capable of acquiring the interference pattern of the entire FPE with a sampling rate of greater than 12 MHz. This optical system utilizes a digital micromirror device to apply a binary mask to the interference pattern of the FPE. The resulting masked image is then rapidly swept across an imaging sensor with a galvanometer, thus encoding time information in the spatial domain. Finally, the PA image is reconstructed using a compressive sensing approach that iteratively solves a convex optimization problem specifically designed for problems where data are under-sampled and the true solution has sparse representation in some related domain (e.g., the gradient domain).

The rest of this paper is organized as follows: In Section 2, we describe the problem and provide the background in compressed sensing needed in our new approach. The results of the simulations are presented in Section 3, with a discussion of these findings and some concluding remarks in Section 4.

## 2. Methods

### 2.1. Optical Setup

**Figure 1:**
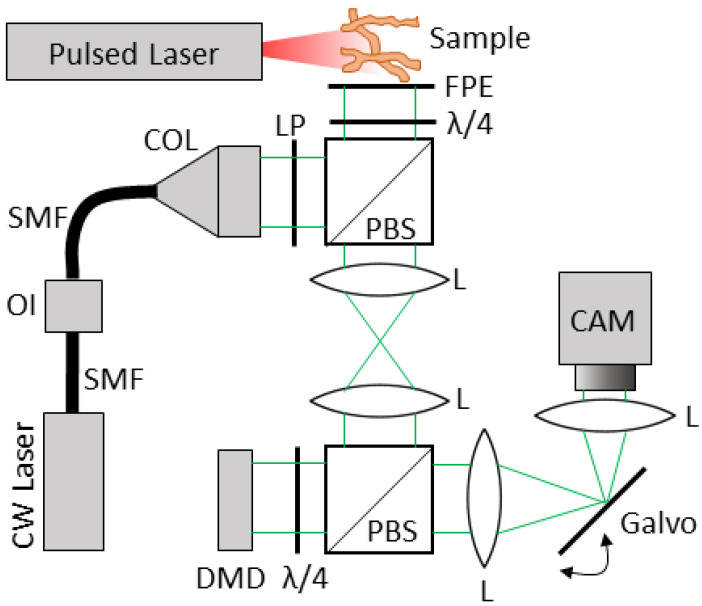
The proposed optical system. SMF = single-mode fiber, OI = optical isolator, COL = collimator, PBS = polarized beam splitter, λ/4 = quarter wave plate, L = lens, CAM = camera, DMD = digital micromirror device, LP = linear polarizer, FPE = Fabry–Pérot etalon.

The proposed optical system is depicted in Figure 1. A pulsed laser is applied to the tissue of interest. Optical absorbers in the tissue then convert the optical energy to heat, inducing a thermoelastic expansion of the surrounding tissue. The expansion generates broadband ultrasound waves, which are detected at the surface of the tissue with a FPE. This interaction of the PA waves with the surface of the FPE results in modulation of the reflected interrogating continuous-wave (CW) laser beam on the opposite side of the FPE. The linear polarizer and quarter wave plates allow for the directed flow of the light through the polarized beam splitters.

The modulated CW laser beam is then imaged onto a digital micromirror device (DMD) via a 4-f optical system, which consists of two lenses spaced by twice their focal length. The DMD consists of a two-dimensional array of mirrors that will either reflect the light along the optical path or deflect it away from it, resulting in a binary mask on the FPE. A second 4-f optical system then images the DMD directly onto an imaging sensor array. A galvanometer is placed in the Fourier plane of the second 4-f system, which rapidly sweeps the image of the masked FPE across the camera during a single exposure.

### 2.2. Continuous Model

We now seek to build a forward model that transforms a given initial pressure distribution (IPD) to a camera image based on the proposed optical system. Let $d,\tau \in R^{+}$. The function P(x,y,z,t) is definedon [0,d]×[0,d]×[0,d]×[0,τ] to be the pressure distribution at (x,y,z) at time *t*. The IPD is then $P_{0}:=P\left(x,y,z,0\right)$. Acoustic waves then propagate outwards in a manner determined by the governing equations

$$\begin{array}{ccc} \frac{\partial \vec{u}}{\partial t} & = & -\frac{1}{\rho_{0}}\nabla P\\ \;\frac{\partial \rho }{\partial t} & = & -\rho_{0}\nabla \cdot \vec{u}\\ P & = & c_{0}^{2}\rho . \end{array}$$

Here $\vec{u}$ is the acoustic particle velocity, ρ is the density, $\rho_{0}$ is the density in the absence of acoustic waves, and $c_{0}$ is the isentropic sound speed [17].

The FPE is placed on the xy-plane and encodes the pressure data P(x,y,0,t). We next define the binary mask $M\subset R^{2}$. The interaction of the light from the FPE with the DMD can be characterized as

(1) $$P_{M}\left(x,y,t\right)=\left\{\begin{array}{cc} P(x,y,0,t) & \mathrm{if}\;(x,y)\in M\\ 0 & \mathrm{else}. \end{array}\right.$$

The data then undergoes a shearing operation from the motion of the galvanometer, leading to

(2) $$P_{S}\left(x,y,t\right)=P_{M}\left(x-\alpha t,y,t\right),$$

where it is assumed that the galvanometer sweeps with a constant speed α>0, where α is determined by the physical limitations of the galvanometer and the focal length of the lenses in the second 4-f system.

Lastly, $P_{S}$ undergoes a temporal integration operation as it is swept across the camera sensor for an exposure time equal to the acoustic wave propagation time τ, yielding the camera image

(3) $$E\left(x,y\right)=\int_{0}^{\tau }P_{S}\left(x,y,t\right)dt=\int_{0}^{\tau }P_{M}\left(x-\alpha t,y,t\right)dt,$$

where $P_{M}\left(x,y,t\right)$ is given in (1). This describes the full continuous forward model.

### 2.3. Discrete Model

We now move to discretize (3) to enable solving the inverse problem. First, the IPD is discretized into a uniform three-dimensional computational grid of size N×N×N for a given choice of N∈Z. The dimension of each voxel is then h×h×h where $h=\frac{d}{N}$. The grid elements are then rearranged to form a single vector *u* of length $N^{3}$. Next, we model the propagation of the acoustic waves through body tissue over time using the k-Wave simulation toolbox in MATLAB [18]. The propagation is also temporally discretized with time steps Δt determined by the Courant–Friedrichs–Lewy condition, which is dependent on $c_{0}$ and *h*. The total number of time steps T∈Z is then calculated as $T=\lfloor \frac{\tau }{\Delta t}\rfloor$. The acoustic waves are observed by the FPE located at the base of the computational grid at each time step. This transformation from the IPD to the sequence of *T* images of size N×N detected by the FPE can be modeled by the $N^{2}T\times N^{3}$ matrix K, which is constructed by simulating the FPE output for each of the standard basis vectors in $R^{N^{3}}$.

The binary mask *M* used in (1) is now discretized to form $M^{\prime }$, and is defined as

(4) $$M_{i,j}^{\prime }=\left\{\begin{array}{cc} 1 & \mathrm{if}\;\left(ih,jh\right)\in M\\ 0 & \mathrm{else}, \end{array}\right.,\;i,j=1,\ldots ,N.$$

The diagonal matrix $M\in R^{N^{2}T\times N^{2}T}$ is subsequently formed by reshaping $M^{\prime }$ into an $N^{2}\times 1$ vector and inserting it into M such that $M_{j+iN^{2},j+iN^{2}}={M^{\prime }}_{j}$ for $j=1,\ldots ,N^{2}$ and i=1,…,(T−1).

The shearing operation is then applied, which we write as matrix S, where the sequence of images is shifted (spatially) along the *x*-axis as a function of time. Since the shearing speed has, in practice, a significantly greater magnitude than Δt, down-sampling is also performed during this step. This is accomplished by calculating the downsize factor $s=\left\lceil \frac{\alpha }{\Delta t}\right\rceil$. For every *s* entries, all but one entry is discarded so that S is a matrix of size $\left(\left\lfloor \frac{T}{s}\right\rfloor +N-1\right)N\left\lfloor \frac{T}{s}\right\rfloor N^{2}\times T$.

Lastly, the light intensity incident to each pixel is summed over time using a left Riemann sum by the $\left(\left\lfloor \frac{T}{s}\right\rfloor +N-1\right)N\times \left(\left\lfloor \frac{T}{s}\right\rfloor +N-1\right)N\left\lfloor \frac{T}{s}\right\rfloor$ matrix I, resulting in the camera image *v*. Since I, S, M, and K are matrices, the full forward model can thus be represented as

**Figure 2:**
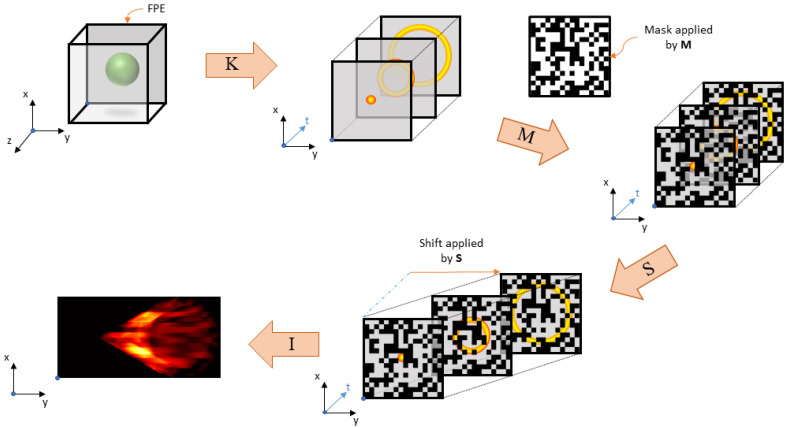
A pictorial representation of the imaging process as individual forward operations.

(5) w=Av+e

where A=ISMK, *w* is a vector of length NL, and *e* is a vector of additive white Gaussian noise with mean zero and covariance matrix $I_{NL}\sigma^{2}$. Although the matrices I, S, and K are not analytically constructed, one can explicitly form A, as will be described in Section 2.5. The parameter *L* is determined by the angular velocity of the galvanometer, and with a constant angular velocity, we have from above that $L=\left\lfloor \frac{T}{s}\right\rfloor +N-1$. Figure 2 indicates how each component of A affects the image.

### 2.4. Image Reconstruction via Compressed Sensing

To reconstruct the IPD, we utilize ideas and algorithms from compressed sensing, which is based on the key notion that a sparse signal can be reconstructed with relatively few measurements. Following the seminal work in [19,20], many investigations have centered around compressed sensing algorithms–sometimes with the goal of generally improving the methodology, e.g., its efficiency, robustness, and accuracy, and in other cases to use the method for a particular application of interest. For example, compressed sensing has been extensively used in the area of PA image reconstruction, [21,22,23,24].

Compressed sensing requires that the image satisfy certain sparsity and incoherence constraints [25]. They are (i) the image should contain only a few nonzero values in some domain, known as the sparse domain, and (ii) the image acquisition domain should not be coherent with the sparse domain. The original image can then be accurately reconstructed from under-sampled data using an iterative method that utilizes a data fidelity term and includes a sparsity constraint.

As we are attempting to reconstruct a vector of length $N^{3}$ with one of length NL, where $L<N^{2}$, this inverse problem is under-determined. Note that in practice, $L\ll N^{2}$, so the compression ratio is quite high. In this work, we arrive at the $L/N^{2}$ ratio of 163/4096≈25. We will address this issue using a compressive sensing approach, which, as noted above, requires that the IPD is sparse in some domain. Since it is anticipated that real-world applications will consider IPDs that are approximately piecewise-constant, the sparsity-enforcing regularization term Φ is chosen as the isotropic discrete total variation (TV) operator. This leads us to the convex optimization problem

(6) $$v=\underset{\hat{v}\geq 0}{arg min}\frac{1}{2}{A\hat{v}-w}_{2}^{2}+\lambda \Phi \left(\hat{v}\right),$$

where A is described in (5), λ is the regularization parameter, and the problem is augmented by the physical constraint v≥0.

Many algorithms have been developed to numerically solve (6), and there have also been numerous investigations into parameter selection [26]. For the simulations we employ the Two-Step Iterative Shrinkage/Thresholding (TwIST) algorithm to recover *v* in (6) [27]. This method brings together the high denoising capabilities of iterative shrinkage/thresholding (IST) and the efficiency for dealing with ill-posed problems of iterative reweighted shrinkage (IRS) algorithms. IST has good denoising properties, while IRS is good at handling ill-posed problems, and TwIST aims at keeping both these advantages. Since the IPD is expected to be nonnegative, we have modified TwIST such that after each iteration, all negative values are set to zero. This modification is not trivial, and further investigation is needed to quantify the stability and accuracy of this revised method. The unmodified TwIST algorithm is commonly used in related applications [12,28].

To compensate for the finite resolution effects and aliasing arising from discretization of the k-Wave simulation, reconstructed IPD is normalized prior to quantitative comparison. We acknowledge that this is not necessarily the best way to treat the error, but the approach is effective in our experiments. More extensive study is required to identify a better mitigating technique. The two main criteria used to quantify the success of the reconstruction are mean square error (MSE) and multi-scale structural similarity (MS-SSIM) index. The MS-SSIM index incorporates image details at different resolutions to provide an image quality assessment based on the human visual system [29]. For MSE, a smaller number indicates less error, while MS-SSIM is between −1 and 1, with 1 indicating a perfect reconstruction.

### 2.5. Simulation Setup and Analysis

In addition to the parameters defined previously, there are several other important parameters to discuss for the simulations. The size of the computational grid for the k-Wave simulation is $N_{c}>N$, where we apply a perfectly matched layer absorbing boundary condition to the edges of the computational grid. The layer occupies a strip of size $N_{L}$ grid points around the outer perimeter of the computational domain. The speed of sound in the medium containing the IPD is $c_{s}$, the length of each voxel is *h*, and the center frequency is *f*.

We now describe how the resulting image is computed from the optical setup given an IPD. An $N_{c}^{3}$ computational grid is created on which to run the k-Wave simulation. The FPE is incorporated as a $N^{2}$ sensor placed parallel to the xy-plane in the extended computational grid with corner at $\left(\frac{N_{c}-N}{2}+1,\frac{N_{c}-N}{2}+1,\frac{N_{c}-N}{2}+1\right)$. The speed of sound is defined corresponding to the average speed of sound in human tissue [30]. The sensor data is stored in a N×N×T array, and we proceed as described in Section 2.4. The parameters chosen for the simulations are defined in Table 1.

To test our new method we will consider (1) the base case cylinder IPD, (2) the base case cylinder IPD rotated so that its axis is parallel to the *x*-axis, and (3) a vessel-like IPD with ten total vessels. The rotated cylinder is included to analyze how the orientation of the cylinder relative to the direction of shearing affects reconstruction. In our simulations, the direction of shearing is parallel to the *x*-axis. In this analysis, the probability that a given pixel, *m*, in the mask is set to one is varied. For this purpose we define

(7) $$\begin{array}{c} p=Prob(m=1). \end{array}$$

The cylinder is passed through the forward model and then added noise to the final image before attempting reconstruction. The regularization parameter in (6) was chosen as $\lambda =2.5\times 10^{-3}$, which was optimized heuristically for the no noise case. The reconstruction is performed for various levels of signal-to-noise ratio (SNR), which is defined as

(8) $$\begin{array}{c} \mathrm{SNR}=20\log_{10}\left(\frac{\mu }{\sigma }\right)\;\mathrm{dB}, \end{array}$$

where μ is the mean of the signal strength in its area of support and σ is the standard deviation of the noise present. Note that μ is approximated over the inferred region of support of the image.

## 3. Results

### 3.1. Baseline Test

**Figure 3:**
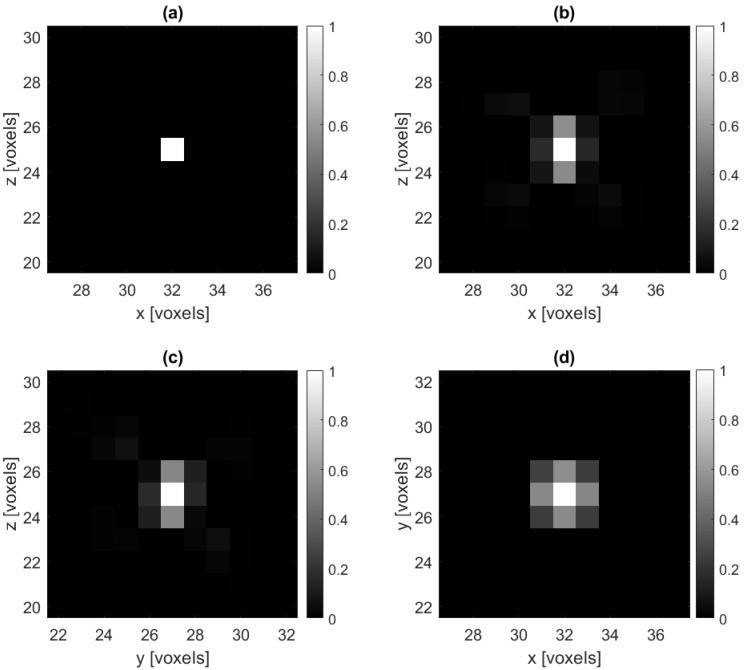
(**a**) A cross-section of the ground truth impulse IPD and (**b**–**d**) cross-sections of a no-noise reconstruction of the impulse IPD. Here we use regularization parameter $\lambda =2.5\times 10^{-4}$ in (6).

As a baseline test, and to demonstrate that our methods perform as expected, Figure 3 displays the reconstruction of an IPD of a single impulse; that is, it contains all zeros except for a single voxel that is set to a value of one. Since the matrix A is constructed by passing each basis vector through the forward model, this is a good test to see if the reconstruction algorithm is working as expected. We see in Figure 3 that, as predicted, the reconstruction of the single impulse is spread across neighboring pixels with a peak at the true impulse pixel.

### 3.2. Simulated Experiments

Having established our method performs as expected in the simple impulse case, we now consider two primary types of more realistic IPDs, namely cylindrical IPDs and vessel-like IPDs, each of which has binary-valued voxels representing either the presence or lack thereof of an initial acoustic impulse response.

**Figure 4:**
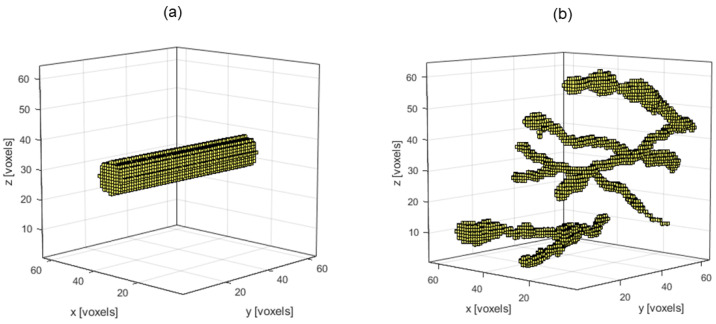
(**a**) The base case cylindrical IPD and (**b**) an example of a vessel-like IPD.

For the base case of the cylindrical IPDs, shown in Figure 4a, a solid cylinder with a radius of six voxels, including the center voxel, and with axis parallel to the y-axis and centered on the xz-plane is used. The vessel-like IPDs, an example of which is displayed in Figure 4b, are constructed with a more random behavior meant to simulate the structure of blood vessels in tissue, including growing and shrinking as well as branching vessels.

**Figure 5:**
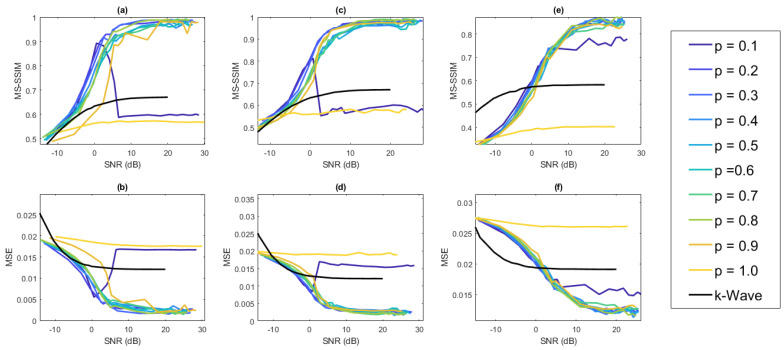
Average MS-SSIM (**a**) and average MSE (**b**) of a cylinder IPD with a radius of six perpendicular to the direction of shearing. Average MS-SSIM (**c**) and average MSE (**d**) of a cylinder IPD with a radius of 6 h parallel to the direction of shearing. Average MS-SSIM (**e**) and average MSE (**f**) of a vessel IPD with ten vessels present. Each point is averaged over five trials for each SNR value considered and is plotted against the SNR value used to calculate the additive Gaussian noise.

In our experiments, we used Gaussian noise with mean zero and variance dependent on the desired SNR parameter given by (8). For each value of SNR examined, the image reconstruction was performed five times (after adding noise as described in Section 2). The average value of MS-SSIM and MSE over those five trials was then computed. Shown in Figure 5 are the MSE and MS-SSIM results for the reconstruction of the cylinder IPD parallel to the y-axis, the cylinder IPD parallel to the x-axis, and the vessel IPD, all for different values of *p* in (7), as well as one using the normalized time reversal reconstruction included with the k-Wave toolbox. The k-Wave reconstruction is performed without compression on the data acquired from the FPE with the appropriate noise added. For SNR greater than 0 dB, we observe that our method performs consistently better than the k-Wave reconstruction for p values between 0.2 and 0.9, and the accuracy of the reconstruction tends to increase faster with our method than with the k-Wave reconstruction as the SNR increases.

**Figure 6:**
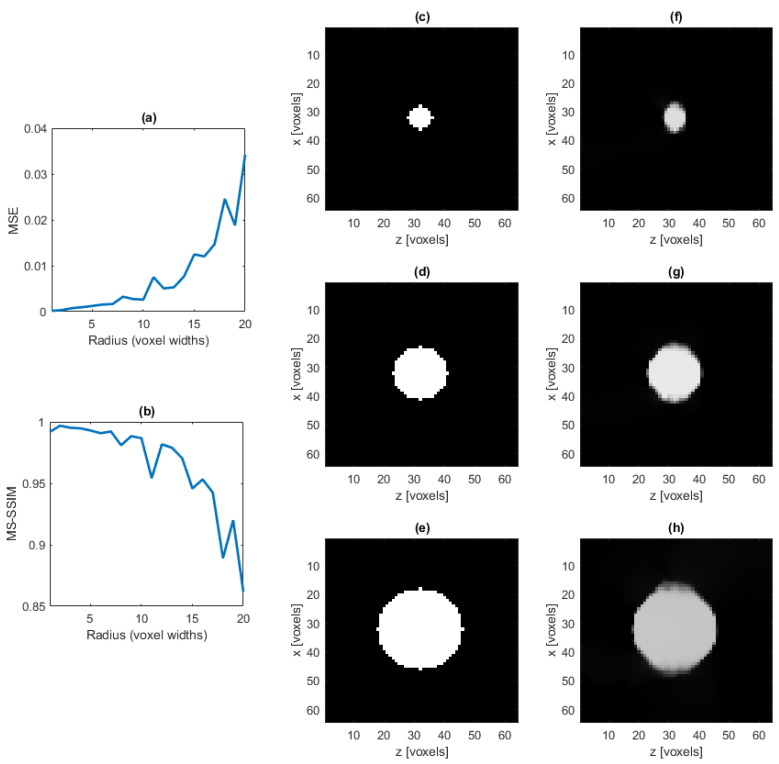
Average MSE (**a**) and average MS-SSIM (**b**) of the reconstructed cylinder IPD with varying radius, averaged over five trials for each radius value considered. A cross section of the ground truth cylinder with radius of five (**c**), ten (**d**) and fifteen (**e**) voxel widths and a cross section of the reconstruction of the same cylinder, respectively, (**f**–**h**). Each cylinder considered is orthogonal to the xz-plane.

We now examine the effects of varying cylinder size on the reconstruction using the base case cylindrical IPD. We fix the amount of noise added so that SNR ≈27 dB and examine the reconstruction using different values for the radius of the cylinder, including the center voxel, in the cylindrical IPD. The results displayed in Figure 5 show comparable performance for p values between 0.2 and 0.8 in (7). The value of p=0.3 was selected for all subsequent analyses. Figure 6 displays the MSE and MS-SSIM results.

**Figure 7:**
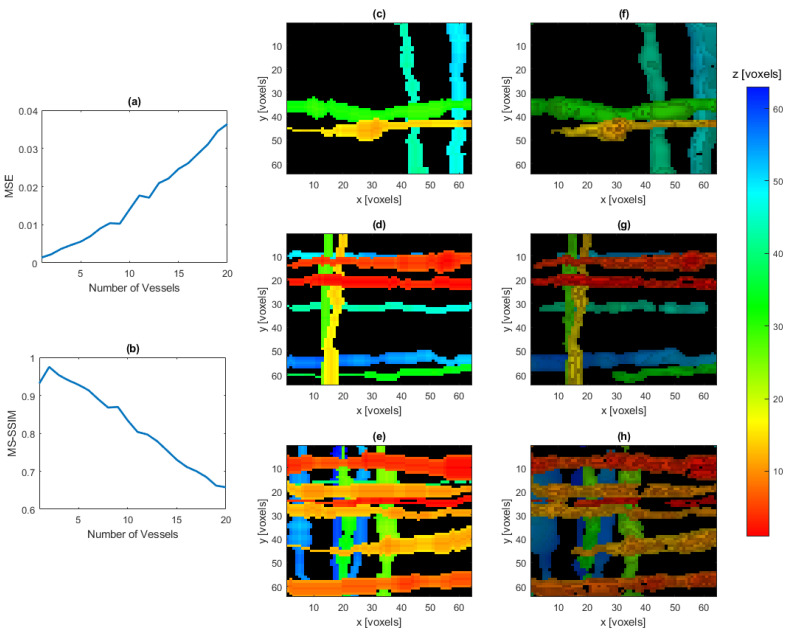
Average MSE (**a**) and average MS-SSIM (**b**) of the reconstructed vessel IPDs with a varying number of vessels present, averaged over twenty IPDs for each number of vessels considered. Ground truth projection onto the xy-plane of a four vessel IPD (**c**), eight vessel IPD (**d**), and twelve vessel IPD (**e**), and the reconstruction of the same IPDs, respectively, (**f**–**h**). In images (**c**–**h**), hue represents depth in the z-dimension, with the colorbar indicating pixel lengths away from the FPE, while intensity is proportional the value of the voxels after being thresholded at 0.15.

We next examine the effect of increased complexity on the reconstruction. The amount of noise is fixed so that SNR ≈25 dB. Using the vessel-like IPDs, the reconstruction is attempted for an increasing number of vessels. These results are displayed in Figure 7.

**Figure 8:**
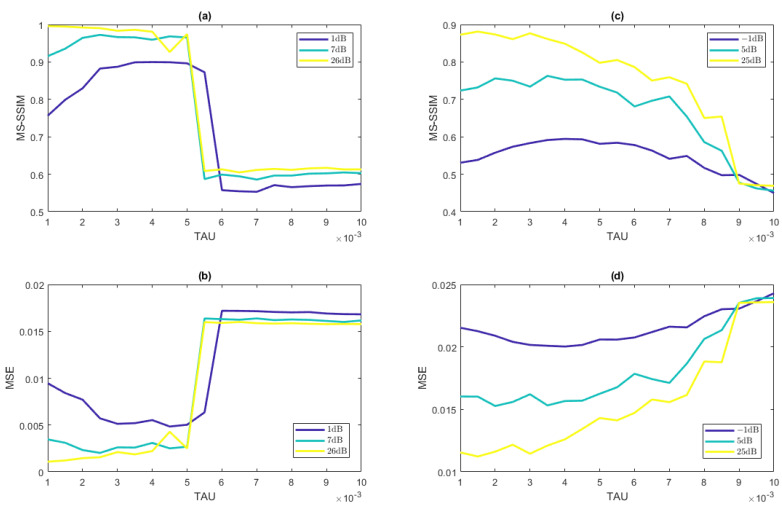
Average MSE (**a**) and average MS-SSIM (**b**) of the reconstructed cylinder IPD as well as average MSE (**c**) and average MS-SSIM (**d**) of the reconstructed vessel-like IPD, averaged over five trials for each value of the regularization parameter considered. Low, medium, and high values of SNR are considered for comparison.

An important consideration in these reconstructions is the choice of regularization parameter, λ in (6). While the optimal regularization parameter is a function of the noise present in the system, it is desirable for the method to be robust in terms of choice of regularization parameter. Figure 8 displays results for the reconstruction of a cylinder orthogonal to the direction of shearing and of a vessel-like IPD with ten vessels present for a range of regularization parameters λ in (6). Observe that our method performs consistently for the same choice of regularization parameter across various noise levels for both the MS-SSIM and MSE metrics. Figure 8 (right) also demonstrates that the method is robust with respect to the choice of regularization parameter for the vessel-like IPD reconstruction. On the other hand, the large jump displayed in Figure 8 (left) shows that the method is not as robust with respect to the choice of the regularization parameter for the single-cylinder case. We speculate that this might be due to the fact that most of the true underlying image has zero value, making it difficult to tune the regularization parameter. We do not see this lack of robustness as a practical issue, however, since real-world applications more closely resemble the multiple vessel case. This issue will be investigated in future work.

## 4. Discussion

In this paper, we modeled a new method for compressed single-shot PA image reconstruction using various types of DMDs to encode temporal information, and then demonstrated through simulated experiments that our approach is capable of accurately reconstructing a variety of IPDs. Moreover, it is robust in the presence of additive Gaussian white noise. We note that while the IPDs modeled here are piecewise constant, the k-Wave toolbox uses methods best-suited for smooth IPDs. We do not anticipate this presenting issues in real-world applications, since the physical process will not experience the aliasing that is observed with the k-Wave simulations.

Figure 5 demonstrates that for a range of probabilities that a given pixel in the binary mask is turned on, i.e., 0.2≤p≤0.8, the performance of our method is consistently better than the k-Wave reconstruction whenever SNR ≥0 dB. This is a significant improvement given that the k-Wave reconstruction is done with the full time-series data and that our method experiences approximately 25-fold compression. While the random construction of the mask was effective in the simulations, there may be other ways to construct the mask leading to more accurate reconstructions in some cases. This will be the subject of future work.

Figure 6 and Figure 7 demonstrate the effectiveness of the reconstruction as the number of nonzero values increases in the system, and we note that we are able to achieve accurate reconstructions in the presence of both large and complex vessel systems.

In future investigations we will assemble the optical system and employ the methods and techniques discussed here to reconstruct phantoms using images generated by the physical forward model. In the construction and implementation of the physical optical system, there are several considerations regarding the continuous wave laser for which to account. In the simulations considered here, the sample was assumed to be under uniform illumination by the laser, while the real system will likely experience a more Gaussian-type illumination. A correction to the forward model would then be needed for spatially varying beam intensity. In addition, the FPE cavity must be tuned to match the wavelength of the continuous wave laser. Nanoscopic variations in cavity thickness could prove detrimental to the sensitivity of the system. Finally, the imaging sensor must be selected to have adequate sensitivity to the continuous wave laser wavelength. As the sweeping speed is increased to reach adequate sampling in time, fewer photons are incident on each camera pixel. Thus, MHz sampling rates may require a sensitive camera and relatively high-power laser.

## Figures and Tables

**Table 1:** The parameters used in our simulations and their associated values.

Parameter	Description	Value
*N*	length of IPD grid [pixels]	64
$N_{c}$	length of computational grid [pixels]	96
$N_{L}$	width of boundary condition layer [pixels]	15
$c_{s}$	speed of sound in the medium [m/s]	1540
*h*	pixel width [μm]	122
*f*	center frequency [MHz]	5
*T*	total time steps	400
Δt	time step size [ns]	24
α	[pixels-widths/s]	12
*s*	downsize factor	4

## Data Availability

All data and code are freely available upon request.

## Abbreviations

The following abbreviations are used in this manuscript:

PA	photoacoustic
FPE	Fabry–Pérot etalon
DMD	digital micro-mirror device
IPD	initial pressure distribution
TwIST	Two-Step Iterative Shrinkage/Thresholding
IST	iterative shrinkage/thresholding
IRS	iterative reweighted shrinkage
MSE	mean square error
MS-SSIM	multi-scale structural similarity
SNR	signal-to-noise ratio
